# Genomic epidemiology of healthcare-associated respiratory virus infections in Pittsburgh, Pennsylvania, 2018–2020

**DOI:** 10.1017/ice.2025.10328

**Published:** 2026-01

**Authors:** Vatsala Rangachar Srinivasa, Marissa P. Griffith, Alexander J. Sundermann, Emma Mills, Nathan J. Raabe, Kady D. Waggle, Kathleen A. Shutt, Tung Phan, Anna F. Wang-Erickson, Graham M. Snyder, Daria Van Tyne, Lora Lee Pless, Lee H. Harrison

**Affiliations:** 1 Microbial Genomic Epidemiology Laboratory, Center for Genomic Epidemiology, University of Pittsburgh, Pittsburgh, PA, USA; 2 Division of Infectious Diseases, University of Pittsburgh School of Medicine, Pittsburgh, PA, USA; 3 Department of Epidemiology, School of Public Health, University of Pittsburgh, Pittsburgh, PA, USA; 4 Department of Infection Control and Hospital Epidemiology, UPMC, Pittsburgh, PA; 5 Department of Pediatrics, Division of Infectious Diseases, University of Pittsburgh School of Medicine, Pittsburgh, PA, USA; 6 Institute of Infection, Inflammation, and Immunity in Children (i4Kids), Pittsburgh, PA, USA; 7 Department of Pathology, University of Pittsburgh School of Medicine, Pittsburgh, PA, USA

## Abstract

**Background:**

Respiratory virus transmission in healthcare settings is not well understood. To investigate the transmission dynamics of common healthcare-associated respiratory virus infections, we performed retrospective whole genome sequencing (WGS) surveillance at three teaching hospitals.

**Methods:**

From January 2, 2018, to January 4, 2020, nasal swab specimens positive for rhinovirus, influenza virus, human metapneumovirus (HMPV), or respiratory syncytial virus (RSV) from patients hospitalized for ≥3 days were sequenced. High-quality genomes were assessed for genetic relatedness using ≤3 single nucleotide polymorphisms (SNPs) as a cutoff, except for rhinovirus (≤10 SNPs). Patient health records were reviewed for genetically related clusters to identify epidemiological connections.

**Results:**

We collected 436 viral specimens from 359 patients: rhinovirus (*n* = 291), influenza virus (*n* = 50), RSV (n = 48), and HMPV (*n* = 47). Of these, 42%% (152/359 patients) were from a pediatric hospital, and 58% were from adult hospitals. WGS was performed on 61.2% (178/291) rhinovirus, 78% (39/50) influenza virus, 90% (43/48) RSV, and all HMPV specimens. Among high-quality genomes, we identified 14 genetically related clusters involving 36 patients (range: 2–5 patients per cluster). We identified common epidemiological links for 53% (19/36) of clustered patients; 63% (12/19) of patients had same-unit stays, 26% (5/19) had overlapping hospital stays, and 11% (2/19) shared common providers. On average, genetically related clusters spanned 16 days (range: 0 − 55 days).

**Conclusion:**

WGS offered new insights into respiratory virus transmission dynamics. These advancements could potentially improve infection prevention and control strategies, leading to enhanced patient safety and healthcare outcomes.

## Introduction

Healthcare-associated respiratory virus infections lead to longer hospital stays, higher morbidity, and increased healthcare resource utilization, such as ICU admissions and mechanical ventilation, leading to higher costs.^1–5^ The patient populations affected include young children, older adults, immunocompromised patients, and those with underlying health conditions, who are at heightened risk of severe respiratory virus infections.^6–8^ Despite this, transmission of common respiratory viruses in hospital settings is understudied, with most previous studies focusing on single-species transmission.^6–12^ This indicates a significant gap in knowledge regarding infection control and patient safety.

Traditional epidemiological methods are commonly used to track and manage the spread of respiratory viruses. These methods involve monitoring for significant increases in infection incidence above baseline levels, which then trigger follow-up investigations.^13–15^ This approach limits the ability to determine outbreaks as soon as a single transmission event, which is necessary for implementing timely interventions and for effectively controlling the spread of the virus early in an outbreak.^16^ Traditional methods also fail to distinguish between a single outbreak or multiple outbreaks of different viral variants.

Recently, the addition of whole genome sequencing (WGS) has augmented traditional outbreak investigation, known as reactive WGS.^8,9,17^ Here, we performed retrospective WGS surveillance of healthcare-associated respiratory viral infections, including rhinovirus, influenza virus, respiratory syncytial virus (RSV), and human metapneumovirus (HMPV), to further understand their transmission dynamics as a pilot study to support future efforts towards implementation of real-time WGS surveillance.

## Methods

### Study setting

This study was performed in three teaching hospitals in Pittsburgh, PA, including two hospitals primarily serving adults and one pediatric hospital (PH), each having a total bed capacity exceeding 700 beds. Ethics approval was obtained from the University of Pittsburgh Institutional Review Board, which included a waiver of informed consent (STUDY21040126).

### Specimen collection

From January 2, 2018, to January 4, 2020, viral transport media of anterior nasal swab specimens positive for respiratory viruses (respiratory viral panel (RVP), GenMark Diagnostics, Carlsbad, CA), including parainfluenza virus, seasonal coronavirus (229E, NL63, OC43, and HKU1), adenovirus, influenza virus, RSV, HMPV, rhinovirus and enterovirus were collected, deidentified and stored at −80°C. Here, we only included rhinovirus, influenza virus, RSV, and HMPV from the three study hospitals because other viruses had insufficient sample sizes to detect meaningful epidemiological links. During this period, respiratory virus testing was done at the discretion of the clinical provider. Specimens were included for inpatients who had been hospitalized for ≥3 days and/or had a recent inpatient or outpatient encounter within 30 days before the positive test date, regardless of the timing or location of symptom onset.

### Specimen processing

Total nucleic acid was extracted using the MagMAX Viral and Pathogen Nucleic Acid Isolation Kit (Thermo Fisher Scientific) per manufacturer’s instructions. Reverse transcription quantitative polymerase chain reaction (RT-qPCR) was performed for all specimens to identify the cycle threshold (Ct) value (**Supp Methods**).^18,19^ Specimens with Ct values ≤30, <32, and ≤39 for rhinovirus, influenza virus, and RSV, respectively, were prepared for WGS.

Sequencing libraries were generated using one of two methods. For all rhinovirus, influenza virus, and RSV specimens with Ct ≥24, a hybridization method was used (respiratory virus research panel, Twist Bioscience).^20^ We modified how individual rhinovirus libraries were combined into one pool prior to the 16-hour hybridization step; these libraries were pooled based on viral copy number instead of equimolar pooling (**Supp Methods**). Each rhinovirus library pool contained eight specimens. Libraries for RSV and influenza virus specimens were pooled at an equimolar concentration based on Ct values (approximately +/−2 SD); each pool contained between four and eight specimens. Additionally, during protocol optimization, eight rhinovirus and two influenza virus specimens with Ct values higher than the cutoff values listed above were sequenced.

For all HMPV specimens, regardless of the Ct value, and for RSV specimens with Ct <24, a tiled PCR amplicon method followed by Illumina DNA Prep was used (**Supp Methods**).^21,22^ For HMPV, apart from the primers described by Tulloch et al.2021, an additional PCR 4 forward primer (5’-GGTCATAAACTCAAAGAAGGTG-3’) was designed to enhance amplification. All libraries were sequenced using either the Illumina MiSeq or NovaSeq X platforms.

### Bioinformatics analyses

Viral species were determined using Kraken2 (v2.1.2),^23^ followed by removal of reads mapping to the human genome. The resulting reads were assembled using the RNA viral SPAdes (v3.15.5) *de novo* approach with default parameters,^24^ except for influenza virus, which was assembled using Iterative Refinement Meta-Assembler (IRMA, v1.0.3).^25^ For viral species other than influenza virus, contigs were then aligned to a reference genome chosen based on nucleotide similarity from a blastn search of publicly available genomes in NCBI; scaffolds were generated using nucmer (v4.0.0).^26^ CheckV (v.1.0.1) was used to estimate the completeness of each *de novo* assembly.^27^ Viral subtypes were determined using NCBI blastn, and genotypes were assigned for rhinovirus.^28^ Genomes passed WGS quality control (QC) if ≥90% of the genome had ≥10× coverage depth and at least 90% estimated completeness from CheckV.

Assembled genomes were aligned using Multiple Alignment using Fast Fourier Transform (MAFFT, v7.515).^29^ We trimmed the 5’ and the 3’ region of the multiple genome alignment to ensure there were no missing bases in those regions and to eliminate sequencing bias due to a drop in sequencing depth in these regions. The resulting alignments were used to identify single nucleotide polymorphisms (SNPs) and to obtain a maximum-likelihood phylogenetic tree using the generalized time-reversible (GTR) approach with FastTree (v0.11.2), with default parameters.^30^ Phylogenetic trees were visualized with iToL.^31^ To identify genetically related clusters, a pairwise SNP analysis using SNP-sites (v2.5.1),^32^ followed by distance-based hierarchical clustering, was performed. Based on our prior experience investigating viral outbreaks in hospital settings, a 3-SNP cutoff with average linkage clustering was chosen to define genetically related clusters of influenza virus, RSV, and HMPV.^33^ To account for the higher mutation rate and greater genetic diversity in rhinovirus,^34^ a 10-SNP cutoff was used for this virus.

### Partial genome analysis

To assess hospital transmission, prior studies have often analyzed partial genomes of influenza virus and rhinovirus.^7–9^ For influenza virus, these studies focused on the variable hemagglutinin (HA) segment; for rhinovirus, the internal ribosomal entry site (IRES) spanning approximately the first 1000 bases of the viral genome are often used. For influenza virus and rhinovirus, we investigated the utility of these regions for transmission if the genomes had >90% coverage at ≥10× depth using a 3- or 2-SNP cutoff for clustering, respectively.

### Epidemiological analyses

We reviewed patient electronic health records (EHR) to identify common epidemiological links among patients in genetically related clusters. We defined a common epidemiological link as one of the following: 1) same hospital unit stay, 2) shared provider, or 3) overlapping hospital stay. The same hospital unit referred to patients sharing the same hospital unit when they tested positive, also implying shared providers. Shared provider referred to patients attended by the same provider while staying in different hospital units. Overlapping stay indicated patients had concomitant stays in different hospital units within the same hospital at the time of testing positive. A 5-day incubation period was applied to all definitions.

## Results

During the study period, we collected 436 rhinovirus (*n* = 291), influenza virus (*n* = 50), HMPV (*n* = 47), and RSV (*n* = 48) specimens from 359 patients (Table 1). Of the 359 unique patient respiratory virus specimens, 152 (42.3%) were from the PH, 124 (34.5%) from adult hospital 1 (AH1), and 83 (23.1%) from adult hospital 2 (AH2). Most patients were male (*n* = 209, 58.2%) and/or white (*n* = 267, 74.4%). There were 48 patients who had more than one respiratory virus specimen collected (range: 2–12; median, 2), and five patients had more than one respiratory virus identified.

**Table 1.** Demographic characteristics of patients with collected respiratory virus specimens

		**Pediatric hospital,** **N (%)**	**Adult hospital 1,** **N (%)**	**Adult hospital 2,** **N (%)**
		***N*** **= 152 (42.3)**	***N*** **= 124 (34.5)**	***N*** **= 83 (23.1)**
**Sex**	**Female**	54 (35.5)	60 (48.4)	36 (43.4)
	**Male**	98 (64.5)	64 (51.6)	47 (56.6)
**Race**	**White**	111 (73)	99 (80)	57 (68.7)
	**Black**	20 (13)	20 (16)	21 (25.3)
	**Other**	6 (4)	–	2 (2.4)
	**Unspecified**	15 (10)	5 (4)	3 (3.6)
**Mean Age**		4.2	55.2	64.3
		Range 14 days–39 years^#^	Range 21–89 years	Range 25–93 years
Respiratory Season*	**2017–2018**	38 (25)	23 (18.6)	27 (32.5)
	**2018–2019**	70 (46)	84 (67.7)	42 (50.6)
	**2019–2020**	44 (29)	17 (13.7)	14 (16.9)
**Organism**	**Rhinovirus/Enterovirus**	116 (76.3)	56 (45.2)	51 (61.5)
	**Influenza A Virus**	3 (2)	28 (22.6)	12 (14.5)
	**Influenza B Virus**	3 (2)	–	4 (4.8)
	**RSV A**	9 (5.9)	–	2 (2.4)
	**RSV B**	11 (7.2)	18 (14.5)	5 (6)
	**HMPV**	10 (6.6)	22 (17.7)	9 (10.8)

* The duration of specimen collection varied each season: 2017–2018: 6 months; 2018–2019: 12 months; 2019–2020: 6 months; ^#^ Adults with complications since childhood continue to receive care at the children’s hospital. Dash indicates the absence of specimens/patients in the specified category.

RSV, respiratory syncytial virus; HMPV, human metapneumovirus.

Of all 436 viral specimens that underwent RT-qPCR, 178 (61.2%) rhinovirus, 39 (78%) influenza virus, and 43 (90%) RSV specimens passed the RT-qPCR QC criteria, respectively (**Supp** Table 1). The median Ct value for rhinovirus specimens was 27.5 (range: 16–41), influenza virus was 26.7 (range: 17–45), RSV was 28.8 (range: 19–45), and HMPV was 30.7 (range: 20–40) (**Supp** Figure 1). We obtained high-quality draft genomes for 114 (64%) rhinovirus/enterovirus, 34 (87%) influenza virus, and 41 (95%) RSV specimens. Additionally, four (50%) rhinovirus and one (50%) influenza virus specimens with high Ct values passed WGS QC. Nearly all (37/38, 97.4%) HMPV specimens with Ct values ≤37 passed WGS QC, suggesting that a higher Ct cutoff could potentially be used to perform WGS on HMPV specimens. RT-qPCR was unable to distinguish between rhinovirus and enterovirus due to high genetic similarity, leading to the inclusion of two enterovirus specimens into our sequencing workflow; however, we identified these genomes using the WGS data and subsequently removed them from further analyses. Two RSV genomes had subtypes that did not match those identified by RT-qPCR and were removed. Additional details are provided in **Supp** Table 5.

**Figure 1.**
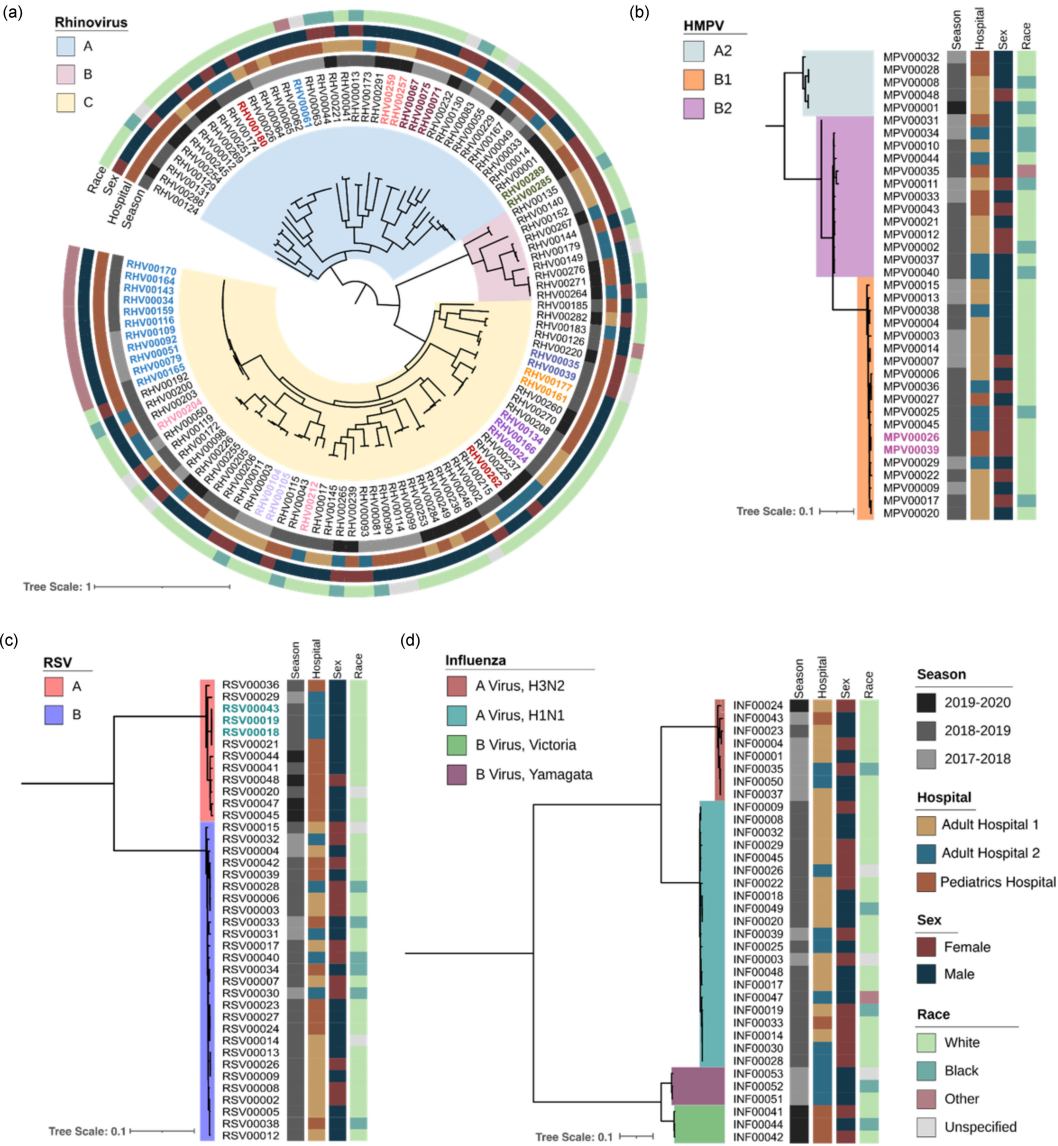
Phylogenetic tree of rhinovirus, human metapneumovirus (HMPV), respiratory syncytial virus (RSV), and influenza virus, by respiratory season, hospital, sex, and race. Tree scale represents the nucleotide substitutions per site. Colored branches represent viral subtypes, colored specimen IDs represent the same-patient specimens, and each vertical strip represents different demographic information.

We next determined the subtypes of rhinovirus, HMPV, and influenza A/B virus from viral genomes that passed WGS QC (Figure 1). Most of rhinovirus genomes belonged to species A (*n* = 65), followed by C (*n* = 41) and B (*n* = 10). We identified 50 distinct rhinovirus genotypes; A38 was most common (*n* = 16), followed by C11 (*n* = 8). For influenza A virus, there were 21 H1N1 and eight H3N2 genomes and, for influenza B virus, there were three genomes each of the Yamagata and Victoria lineages. For HMPV, most genomes belonged to subtype B1 (*n* = 20), followed by B2 (*n* = 12), and A2 (*n* = 5).

**Figure 2.**
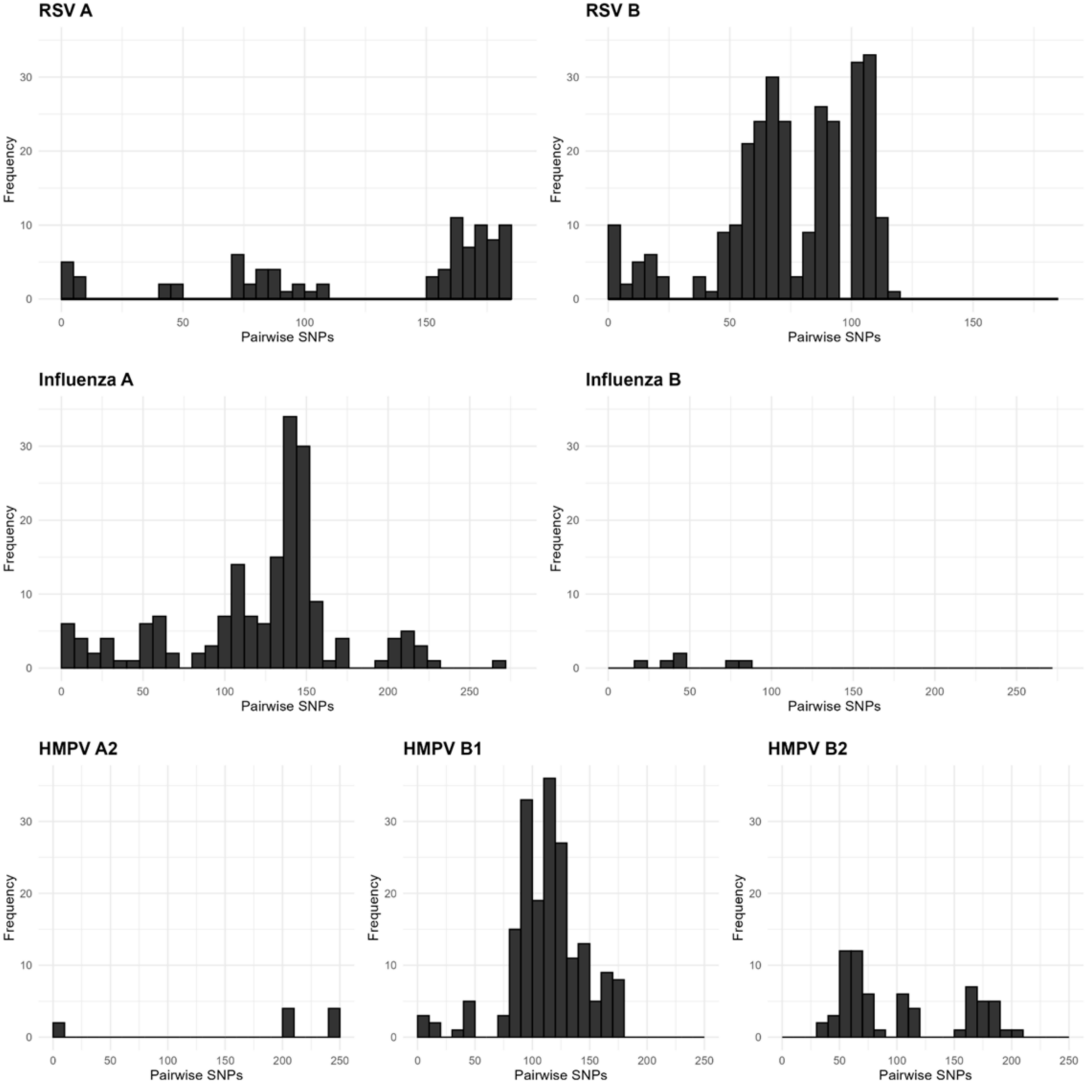
Pairwise single nucleotide polymorphism (SNP) distributions for RSV (respiratory syncytial virus), influenza virus, and HMPV (human metapneumovirus) genomes. Pairwise SNPs were assessed for all genomes in a given viral subtype, and histograms show the distribution of pairwise SNP distances.

**Figure 3.**
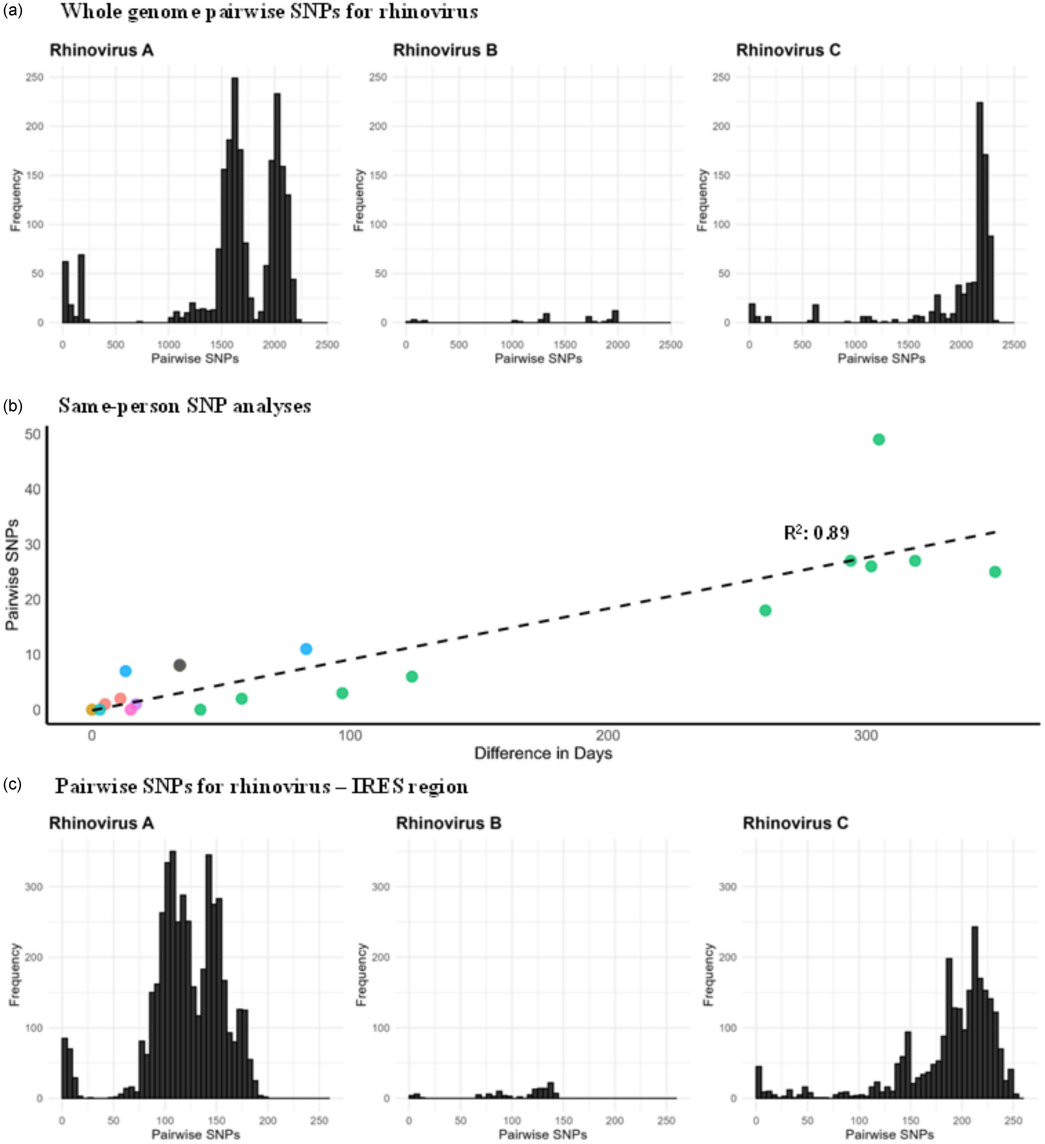
**3a.** Whole genome pairwise SNP distributions for different rhinovirus species; **3b.** Pairwise SNPs versus days between rhinovirus specimens collected from the same patients and belonging to the same genotype. Circles of the same color represent individual patients; **3c.** Pairwise SNP distributions for the rhinovirus IRES region for different rhinovirus species.

We next assessed whether there was sufficient genetic diversity to determine if WGS could identify putative transmission events. The same-subtype pairwise SNPs for RSV ranged between 0–186 (median = 91), HMPV ranged between 0–250 (median = 114), and influenza virus within the same subtype ranged between 0–277 (median = 136; Figure 2). Rhinovirus had the widest range of pairwise SNPs among genomes belonging to the same species, ranging from 0–2305 (median = 1934; Figure 3a). These data suggest that the viral populations sampled were genetically diverse and, therefore, appropriate for the analysis of closely related genomes.

A total of 29 rhinovirus genomes from nine patients were obtained through repeated sampling of the same patient (2–11 genomes per patient). Pairwise SNP analysis of these same-person, same-species genomes were closely related (median: 6, range: 0–49 SNPs), with SNPs increasing as the time between specimen collection advanced (Figure 3b). In one patient, two rhinovirus A specimens that were collected 10 days apart differed by 1,541 SNPs, indicating co-infection with different rhinovirus A strains (A38 and A82). For rhinovirus, 28% of the SNPs within the same patient were in the non-coding (intergenic) regions and 72% were in the coding region of the polyprotein. Among those in the coding region, 47% were non-synonymous mutations and 53% were synonymous mutations. There were two HMPV specimens from the same patient, collected 45 days apart and differed by 1 SNP, which resulted from a non-synonymous mutation in the F gene (fusion protein). In contrast, three RSV specimens from one patient collected over 31 days were genetically identical. Together, these data demonstrate the ability to identify both co-infection and prolonged infection using WGS data.

We next explored using partial genomes for influenza virus and rhinovirus to assess putative transmission, as previously published.^7–9^ For influenza virus, with a 3 SNP cutoff for the HA segment, the whole genome SNPs ranged from 0–34. For rhinovirus, pairwise SNPs in the IRES region ranged from 0–254 (Figure 3c). A 0 SNP cutoff in the IRES region corresponded to 0–52 SNPs in the whole genome, while a 1 SNP cutoff corresponded to 7–63 SNPs. These findings suggest that using only the HA segment for influenza virus and IRES for rhinovirus to define transmission was inadequate.

**Figure 4.**
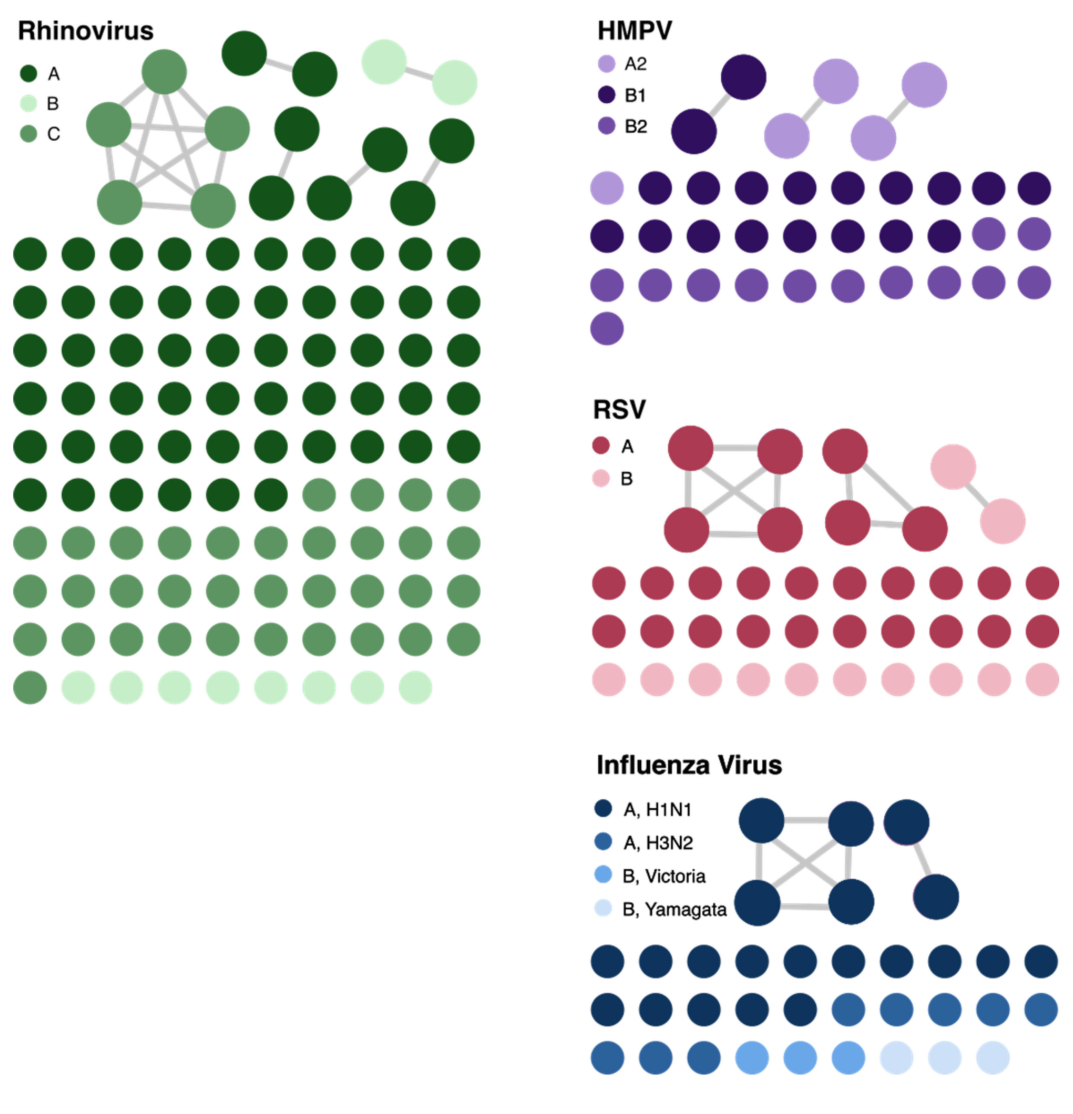
Cluster networks of respiratory virus genomes analyzed for putative transmission. The different color gradients within each virus represent different subtypes of the virus. The connected circles show patient specimens that were genetically related as defined by cutoffs described in the Methods section. The network plot was visualized with Gephi. RSV, respiratory syncytial virus; HMPV, human metapneumovirus.

**Figure 5.**
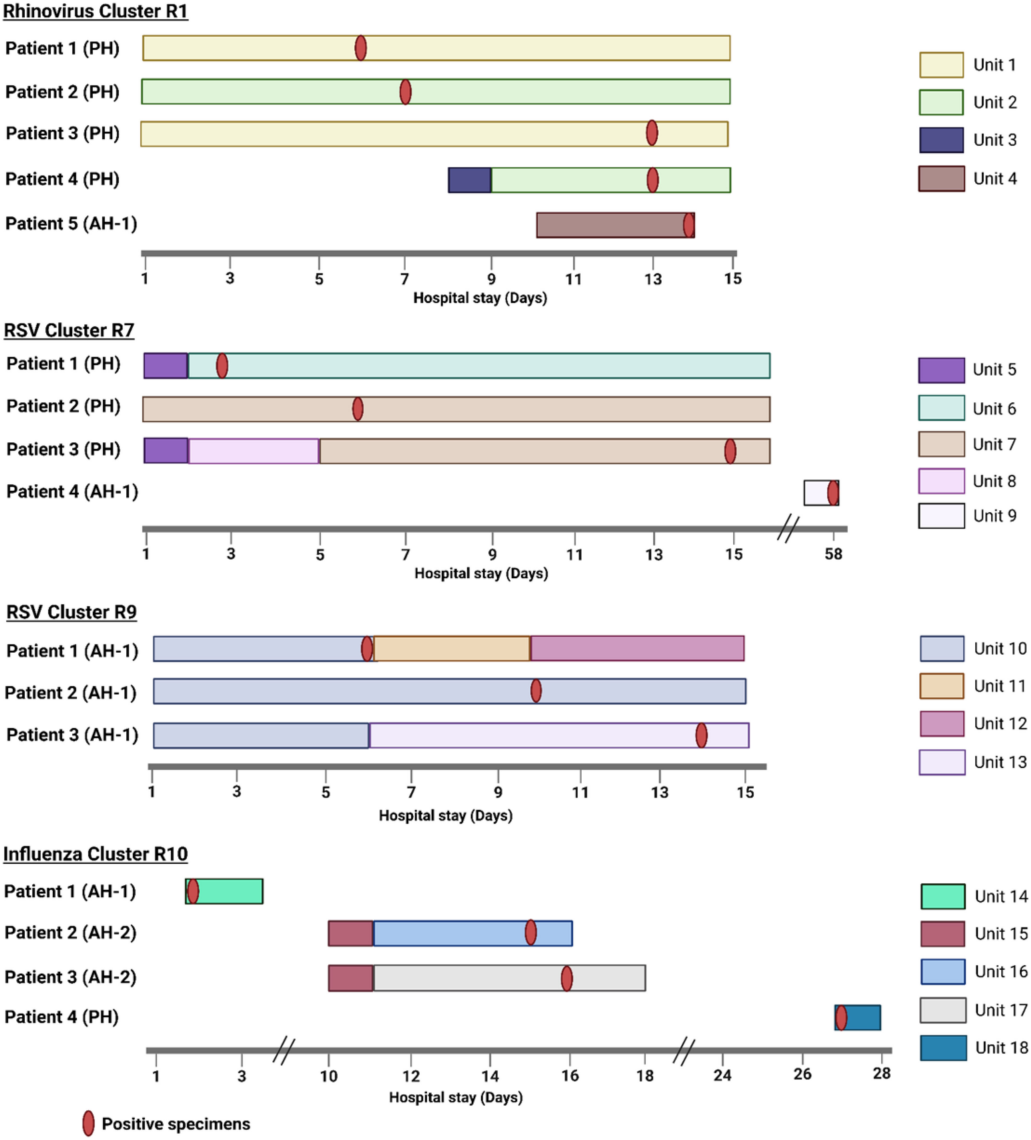
Presumed transmission patterns of genetically related respiratory virus clusters with >2 patients. Day 1 on x-axis is 5 days before the first positive test date within a cluster, unless the specimen was collected on the first day of admission. Created in BioRender. Rangachar Srinivasa, V. (2025) https://BioRender.com/mdnle2e.

We identified 14 genetically related clusters containing 36 patients, ranging from 2–5 patients per cluster (Table 2); no patients were involved in more than one cluster. Overall, 36/359 (10%) patients were included in a transmission cluster. Rhinovirus genomes formed six clusters, followed by RSV and HMPV with three clusters each, and two clusters of influenza virus (Figure 4). We identified common epidemiological links in 19/36 (53%) patients. Of these, 12/19 (63%) patients shared the same unit prior to or at the time they tested positive, 5/19 (26%) had an overlapping hospital stay, and 2/19 (11%) shared a healthcare provider. As a more detailed example, RSV cluster 7 (Figure 5 **)** showed two distinct possibilities of transmission. First, patients 1 and 3 might have been exposed to RSV in unit 5, with the possibility of patient 3 having a longer incubation period, transmitting to patient 2 on a separate unit (unit 7). Second, patients 1 and 2 could have had out-of-unit exposure, with patient 2 transmitting the virus to patient 3 during the same-unit stay (unit 7). Additionally, a fourth patient at a different hospital was also in the cluster, suggesting complex transmission dynamics. In RSV cluster 9, all three patients had an overlapping same-unit stay (unit 10), indicating likely exposure from that location. Patients from multiple hospitals were identified in 9/14 (64%) clusters. On average, the duration of genetically related clusters was 16 days, ranging from 0–55 days.

**Table 2.** Summary of genetically related clusters

**Cluster**	**No.** **Patients**	**Hospital**	**Epidemiologic link**	**Virus**	**Time between first and last specimen (days)**
1	5	PH (*n* = 4) AH-1 (*n* = 1)	PH: Overlapping stay	Rhinovirus C	8
2	2	PH	Shared unit	Rhinovirus A	12
3	2	PH (*n* = 1) AH-2 (*n* = 1)	Unknown	Rhinovirus A	12
4	2	PH (*n* = 1) AH-2 (*n* = 1)	Unknown	Rhinovirus A	0
5	2	PH (*n* = 1) AH-1 (*n* = 1)	Unknown	Rhinovirus A	2
6	2	PH (n = 1) AH-2 (n = 1)	Unknown	Rhinovirus B	5
7	4	PH (*n* = 3) AH-1 (*n* = 1)	PH: Shared unit AH-1: Unknown	RSV B	55
8	2	PH (*n* = 1) AH-2 (*n* = 1)	Unknown	RSV A	33
9	3	AH-1	Shared unit	RSV B	8
10	4	PH (*n* = 1) AH-1 (*n* = 1) AH-2 (*n* = 2)	AH-2: Shared unit PH/AH-1: Unknown	Influenza A virus	25
11	2	AH-1	Shared unit	Influenza A virus	4
12	2	PH (*n* = 1) AH-1 (*n* = 1)	Unknown	HMPV A2	0
13	2	AH-1	Unknown	HMPV A2	32
14	2	PH	Common provider	HMPV B1	9

PH, Pediatric hospital; AH-1, Adult hospital 1; AH-2, Adult hospital 2; RSV, respiratory syncytial virus; HMPV, human metapneumovirus

## Discussion

In this study, we demonstrated that WGS surveillance could provide valuable insights into the transmission patterns of rhinovirus, influenza virus, RSV, and HMPV in three Pittsburgh hospitals. This study adds to the existing literature by using systematic WGS surveillance for healthcare-associated infections of four different respiratory viruses. We successfully sequenced 74% of the genomes that passed the RT-qPCR Ct criteria, identifying 14 genetically related clusters of respiratory viruses containing 36 patients. The overall proportion of patients in a transmission cluster was 10%. A plausible transmission route was identified for 53% of these patients.

Conversely, 47% of patients with genetically related genomes had no identifiable epidemiological links. It is possible that healthcare workers (HCWs) or visitors were a source of transmission; thus, we were unable to identify the link because we did not have these specimens to sequence. Our inability to identify a common link could be because there simply was no epidemiologic connection. At the time of this study, facilities encouraged respiratory etiquette, symptom monitoring, and vaccination (mandatory for HCWs unless medically or religiously exempt). In response to isolated clusters, hospital unit-based masking may have been encouraged or required. However, there was no requirement for regular asymptomatic screening of HCWs or universal masking, so we could not determine if patients were in contact with respiratory virus positive HCWs. It is also possible that some of these patients might have been in direct contact with each other in common hospital spaces, such as the cafeteria or the play area in the PH. Additionally, 64% of clusters included patients from multiple hospitals, suggesting the possibility of community transmission, such as transmission at common gatherings, places of employment, daycare centers, or similar settings.^35^ Furthermore, this could also be due to under-diagnosis of respiratory infections and non-sequenced specimens. Additionally, during periods of high respiratory virus incidence, some cases classified as healthcare-associated may have reflected strains that were widely circulating in the community. Investigating these additional scenarios was beyond the scope of this study.

The average cluster duration was 16 days, ranging between 0–55 days. This could suggest either the possibility of longer contagious periods and possible asymptomatic transmission or gaps in the transmission chain due to specimens that were not sequenced. Future investigations should focus on assessing the duration of contact and droplet precautions followed in hospitals for patients with respiratory virus infections. These long-spanning clusters would be undetected via traditional epidemiological investigations, highlighting the importance of WGS surveillance in identifying cryptic transmission.

Interestingly, one immunocompromised patient sampled in this study tested positive for rhinovirus for approximately one year (Figure 3b, green data points). One of the rhinovirus genomes sequenced from this patient, collected 16 days after the initial positive swab, was genetically related to viruses sampled from other patients. This suggests prolonged infection with intra-host viral evolution and transmission to other patients, consistent with other reports of immunocompromised patients with prolonged respiratory virus shedding serving as a source of transmission.^36–38^

Relying on the genetic relatedness of only the HA segment for influenza virus and the IRES region for rhinovirus has been a common practice for identifying influenza virus and rhinovirus transmission.^7–9^ Our analyses showed that the partial genome SNPs for these viruses underestimated the genome-wide SNPs, resulting in overcalling transmission, and was insufficient to confirm or refute transmission. Furthermore, a 10 SNP cutoff was used for whole genome analyses of rhinovirus transmission. However, no epidemiological links were found beyond a 3 SNP difference, suggesting that this cutoff might be sufficient for identifying rhinovirus transmission clusters.

Our study has several limitations. We were unable to sequence all the collected specimens because some did not pass sequencing QC criteria. We might have underestimated the number of transmission clusters due to our inclusion criteria and/or the arbitrary SNP cutoffs used. We might have missed identifying common epidemiological links, such as a shared provider between two patients, due to limitations associated with the EHR review. Also, not every HCW-patient encounter was reported in the EHR. The generalizability of our findings may be limited due to our inclusion criteria, specific hospital setting and policies, and variations in clinical criteria for testing. As this was a retrospective study, we could not evaluate the implications of our findings on infection prevention and control practices. Additionally, this study did not capture any changes made since the SARS-CoV-2 pandemic. This makes it difficult to determine whether real-time WGS surveillance of respiratory viruses would be useful in reducing transmission.

In conclusion, the integration of WGS surveillance and EHR review elucidated respiratory virus transmission patterns across three hospitals. While this approach shows potential for detecting healthcare-associated transmissions of respiratory viruses, its broader implications warrant additional investigation. These advancements have the potential to improve infection prevention and control strategies, leading to enhanced patient safety and healthcare outcomes. Future studies should focus on performing real-time WGS surveillance of respiratory viruses to determine the potential utility and feasibility of this approach for routine infection prevention and control practice.

## Supporting information

Rangachar Srinivasa et al. supplementary material 1 Rangachar Srinivasa et al. supplementary material

Rangachar Srinivasa et al. supplementary material 2 Rangachar Srinivasa et al. supplementary material

Rangachar Srinivasa et al. supplementary material 3 Rangachar Srinivasa et al. supplementary material

## Data Availability

The NCBI, GISAID, and SRA accession numbers for the respiratory viral genomes used in this study can be found in **Supp** Table 5. Sequence data can be found at NCBI BioProject PRJNA1301846.

## Supplementary material

To view supplementary material for this article, please visit https://doi.org/10.1017/ice.2025.10328
