# Supplementary figures and images for: Genomic epidemiology of healthcare-associated respiratory virus infections in Pittsburgh, Pennsylvania, 2018–2020
Source: Infect Control Hosp Epidemiol. 2025 Nov 4;47(1):53–63. doi: 10.1017/ice.2025.10328 (PMC12780897; doi:10.1017/ice.2025.10328)

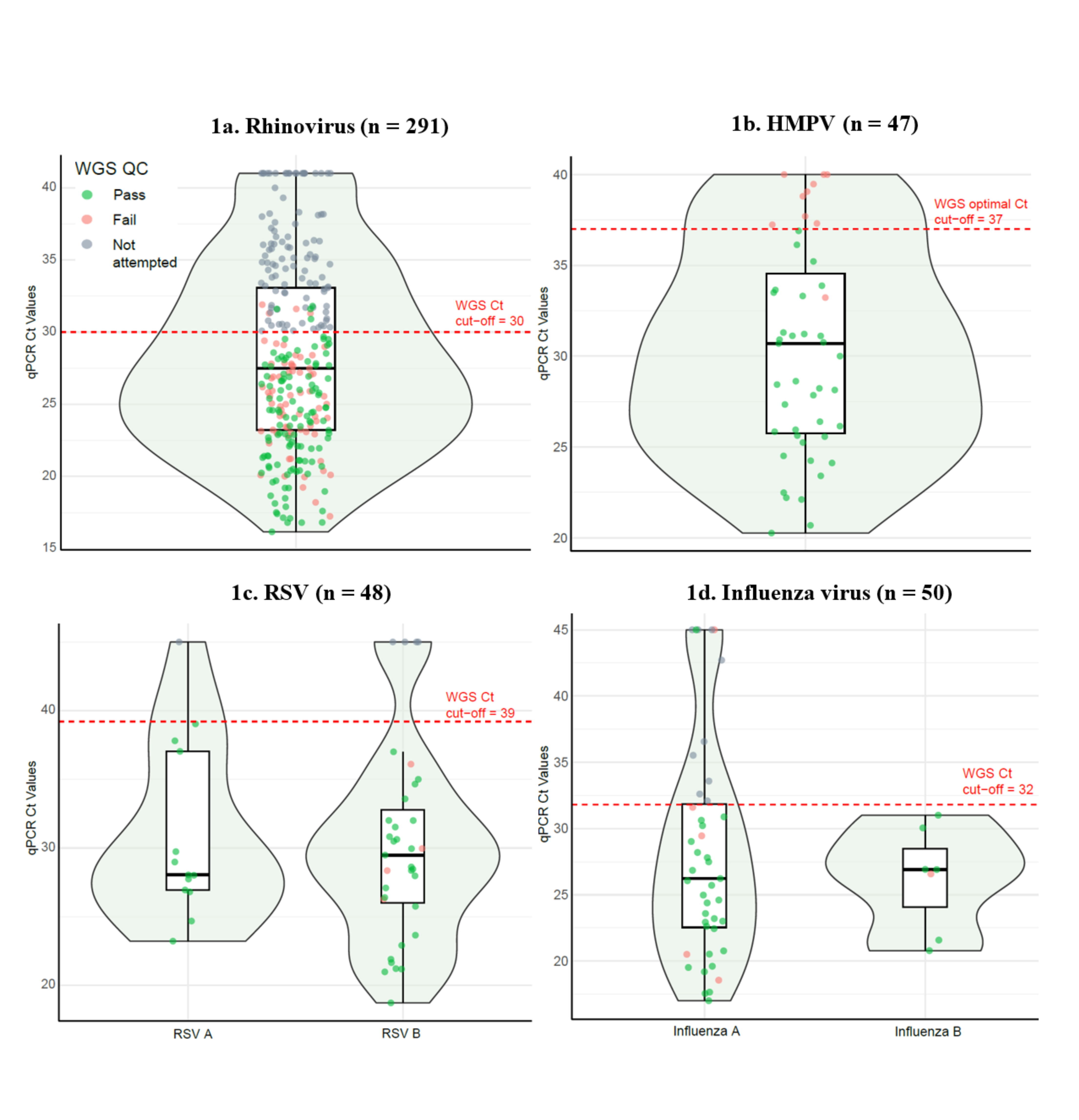

Supplement: Rangachar Srinivasa et al. supplementary material 1 — Rangachar Srinivasa et al. supplementary material [file S0899823X25103280sup001.tiff]
