# Supplementary material for: Genomic epidemiology of healthcare-associated respiratory virus infections in Pittsburgh, Pennsylvania, 2018–2020
Source: Infect Control Hosp Epidemiol. 2025 Nov 4;47(1):53–63. doi: 10.1017/ice.2025.10328 (PMC12780897; doi:10.1017/ice.2025.10328)
Supplement: Rangachar Srinivasa et al. supplementary material 3 — Rangachar Srinivasa et al. supplementary material [file S0899823X25103280sup003.docx]

**Supplementary material**

**Supplementary Table 1.** Overview of whole genome sequencing (WGS) metrics

| **Metrics/virus** | **Rhinovirus** | **Influenza A/B virus** | **RSV A/B** | **HMPV** |
| --- | --- | --- | --- | --- |
| **Collected** | 291 specimens | 50 specimens | 48 specimens | 47 specimens |
| **Passed RT-qPCR QC** | 178/291 specimens (61.2%) | 39/50 specimens (78%) | 43/48 specimens (90%) | ─ |
| **Passed WGS QC** | 114/178 genomes  (64%) | 34/39 genomes  (87%) | 41/43 genomes  (95%) | 37/47 genomes  (79%) |
| **Failed species QC** | 2 genomes | ─ | 2 genomes | ─ |
| **High Ct genomes, passed WGS QC*** | 4/8 genomes  (50%) | 1/2 genomes  (50%) | ─ | ─ |
| **Total genomes included (whole genome analyses)** | 116 genomes | 35 genomes | 39 genomes | 37 genomes |
| **Genetically related** | 15 patients  (6 clusters) | 6 patients  (2 clusters) | 9 patients  (3 clusters) | 6 patients  (3 clusters) |
| **Epidemiologically linked** | 7/15 patients  (47%) | 4/6 patients  (67%) | 6/9 patients  (67%) | 2/6 patients  (33%) |

* While optimizing the protocols for whole genome sequencing, we sequenced a few high Cycle threshold (Ct) specimens of rhinovirus and influenza virus. Dash indicates the absence of respiratory viral specimens/genomes in the specified category.

RSV, respiratory syncytial virus; HMPV, human metapneumovirus

**Supplementary Methods**

Reverse transcription quantitative polymerase chain reaction (RT-qPCR)

To determine the cycle threshold (Ct) value, RT-qPCR was performed for all respiratory virus specimens using 7500 Fast Real-Time PCR system (Applied Biosystems). The assay was performed on technical duplicates using the extracted viral nucleic acid, except for rhinovirus, for which cDNA was used as a template. Molecular grade, nuclease-free water was included as a no template control. Details of RT-qPCR for each virus are described below. For all viral specimens, RT-qPCR data was analyzed by manually adjusting the threshold to approximately three-quarters of the log-exponential phase.

*Influenza virus*

The CDC’s influenza/SARS-CoV-2 multiplex assay was utilized to determine the Ct values for influenza A/B virus and included the seasonal influenza positive template control (SIPC) prepared per CDC protocol [1]. Each RT-qPCR reaction was prepared by combining 6.5µl of TaqPath 1-Step Multiplex Master Mix (no rox), 3µl each of primer and probe mix, 7.75µl of molecular grade, nuclease-free water, and 5µl of the extracted viral nucleic acid. The primers and probes used in this assay are detailed in **Supp Table 2a**. The thermal cycling conditions were as follows: incubation at 25^o^C for 2 min, reverse transcription at 50^o^C for 15 min, followed by *Taq* activation at 95^o^C for 2 min, and then 45 cycles of 95^o^C for 15 sec and 55^o^C for 30 sec.

**Supp Table 2a.** Forward (For) and reverse (Rev) primer and probe sequences used for influenza virus RT-qPCR assay. Note, SC2 refers to SARS-CoV-2 and was included in the primer pool as part of the CDC protocol. A positive template control was used to amplify human ribonuclease P RNA (RP).

| **Name** | **Description** | **Oligonucleotide Sequence (5’ – 3’)** | **Concentration** |
| --- | --- | --- | --- |
| **InfA-F** | InfA For1 | CAA GAC CAA TCY TGT CAC CTC TGA C | 3.33 µM |
|  | InfA For2 | CAA GAC CAA TYC TGT CAC CTY TGA C | 3.33 µM |
| **InfA-R** | InfA Rev1 | GCA TTY TGG ACA AAV CGT CTA CG | 5.00 µM |
|  | InfA Rev2 | GCA TTT TGG ATA AAG CGT CTA CG | 1.67 µM |
| **InfB-F** | InfB For | TCC TCA AYT CAC TCT TCG AGC G | 6.67 µM |
| **InfB-R** | InfB Rev | CGG TGC TCT TGA CCA AAT TGG | 6.67 µM |
| **SC2-F** | SC2 For | CTG CAG ATT TGG ATG ATT TCT CC | 6.67 µM |
| **SC2-R** | SC2 Rev | CCT TGT GTG GTC TGC ATG AGT TTA G | 6.67 µM |
| **RP-F** | RNase P For | AGA TTT GGA CCT GCG AGC G | 6.67 µM |
| **RP-R** | RNase P Rev | GAG CGG CTG TCT CCA CAA GT | 6.67 µM |
| **InfA-P** | [InfA probe](https://www.cdc.gov/coronavirus/2019-ncov/lab/multiplex.html#anchor_60494) | FAM/TGC AGT CCT **/**ZEN**/**CGC TCA CTG GGC ACG/3IABkFQ | 1.67 µM |
| **InfB-P** | [InfB Probe](https://www.cdc.gov/coronavirus/2019-ncov/lab/multiplex.html#anchor_60494) | YakYel/CCA ATT CGA/ZEN/ GCA GCT GAA ACT GCG GTG/3IABkFQ | 1.67 µM |
| **SC2-P** | [SC2 Probe](https://www.cdc.gov/coronavirus/2019-ncov/lab/multiplex.html#anchor_60494) | TexRd-XN/ATT GCA ACA/TAO/ ATC CAT GAG CAG TGC TGA CTC/3IAbRQSp | 1.67 µM |
| **RP-P** | [RNase P Probe](https://www.cdc.gov/coronavirus/2019-ncov/lab/multiplex.html#anchor_60494) | CY5/TTC TGA CCT /TAO/ GAA GGC TCT GCG CG/3IAbRQSp | 1.67 µM |

*RSV and HMPV*

The TaqMan microbe detection assay (ThermoFisher Scientific) was used to determine the Ct values for RSV A (assay ID: Vi99990014_po), RSV B (assay ID: Vi99990015_po) and HMPV (assay ID: Vi99990004_po). A 1:10 dilution of [TaqMan™ Respiratory Tract Microbiota Amplification Control](https://www.thermofisher.com/order/catalog/product/A39178) (ThermoFisher Scientific) was used as a positive control template. Each RT-qPCR reaction was prepared by combining 5µl of 4× TaqPath 1-Step RT-qPCR master mix, 1µl of 20× primer/probe mix, 11.5µl of molecular grade nuclease-free water, and 2.5µl of extracted viral nucleic acid. The thermal cycling conditions were as follows: incubation at 25^o^C for 2 min, reverse transcription at 50^o^C for 15 min, followed by *Taq* activation at 95^o^C for 2 min, and then 40 cycles of 95^o^C for 3sec and 60^o^C for 30sec.

*Rhinovirus*

To determine the Ct value of rhinovirus specimens, we performed a two-step RT-qPCR protocol as described in Ng. et al., 2016 [2]. First and second strand cDNA were synthesized from the extracted viral nucleic acid using Protoscript II First Strand cDNA Synthesis and the NEBNext Ultra II Non-Directional RNA Second Strand Synthesis kits (New England Biolabs). This was followed by SYBR green PCR amplification using primers specific to rhinovirus (**Supp Table 2b**). Human rhinovirus 77 strain 130-63 (ATCC) was used as positive control. Each reaction was prepared by combining 10µl of 2× SYBR green master mix, 1.8µl of each forward and reverse primers, 4.4µl of molecular grade, nuclease-free water, and 2µl of cDNA. The thermal cycling conditions were as follows: incubation at 95^o^C for 2 min, followed by 40 cycles of 95^o^C for 3sec and 60^o^C for 30sec.

**Supp Table 2b.** Primers used for rhinovirus RT-qPCR as described in Ng. et al., 2016.

| Primer name | Primer sequence (5' – 3') |
| --- | --- |
| qR447 forward | GGCCCCTGAATGYGGCTAA |
| qR561 reverse | GAAACACGGACACCCAAAGTAG |
| R529 probe | AYGGRACCRACTACTTTG |

Copy number assay for Rhinovirus – for equimolar pooling of libraries

The manufacturer's protocol for pooling specimens was to combine eight specimens into a single sequencing pool; however, we observed a notable difference in the number of reads per specimen in a given pool, where a specimen with a minimal to modest difference in Ct value to another specimen would result in an imbalance in the number of reads following sequencing. To overcome this limitation, we modified the protocol to pool specimens by copy number, instead of equimolar pooling. To quantify the viral copy number of the clinical rhinovirus specimens, we constructed a standard curve by serially diluting RNA obtained from a reference sample (quantitative genomic RNA from human rhinovirus 77 strain 130-63; ATCC; serial dilutions spanned 20×10^3^ to 20×10^-1^ copies/μl). A two-step RT-qPCR protocol was performed on these serially diluted standards as previously described. The resulting Ct values were used to construct a standard curve, which was utilized to determine the viral load of each clinical specimen (**Supp Table 3**).

| **Quantity (copies/**μ**l)** | **Cycle threshold (Ct)** |
| --- | --- |
| 20000 (20 ×10^3^) | 21.0 |
| 2000 (20 ×10^2^) | 24.3 |
| 200 (20 ×10^1^) | 27.6 |
| 20 (20 ×10^0^) | 31.8 |
| 2 (20 ×10^-1^) | 35.3 |

**Supp Table 3.** Copy number calculation for rhinovirus

Human metapneumovirus (HMPV) and respiratory syncytial virus (RSV) sequencing protocol modifications.

For HMPV cDNA synthesis and genome amplification, we used a tiled single-plex PCR amplicon approach as described by Tulloch et al., 2021 that was modified to amplify the viral genomes prior to preparing the specimens for sequencing [3]. The cDNA synthesis was performed using SuperScript IV VILO Master Mix per manufacturer's protocol (Invitrogen, Carlsbad, CA, USA). The cDNA was divided into four tiled, long-range PCR reactions, each separately amplifying one section of the HMPV genome. PCR amplification was performed using Platinum™ SuperFi™ PCR Master Mix per manufacturer's protocol (Invitrogen). Apart from the primers mentioned in Tulloch et al. (**Supp Table 4**), an additional PCR 4 forward primer (5'-GGTCATAAACTCAAAGAAGGTG-3') was designed to enhance amplification for some specimens that failed using the originally published primer set.

Similar to HMPV, a tiled amplicon approach was used to amplify the RSV genome. A one-step reaction including cDNA synthesis and PCR amplification was used according to Dong et al., 2023 [4]. Six amplicons were combined into two primer pools that amplified the viral genome (**Supp Table 4**). The extracted total nucleic acid was divided into two reactions, one for each primer pool. We used SuperScript IV One-Step RT-PCR (Invitrogen) for both cDNA synthesis and PCR amplification.

For both HMPV and RSV, the resulting PCR product was visualized for quantification and to confirm amplification at the expected fragment size using the D5000 reagents on Agilent 4200 TapeStation system per manufacturer's protocol. Specimens that successfully amplified were pooled into one tube to a final volume of 40μl; we combined each pool based on concentration to ensure even sequencing coverage across the entire genome. The pooled specimens were purified using 1.8× AMPure XP beads and eluted to 55μl final volume (Beckman Coulter, Pasadena, CA, USA). Specimens that failed to amplify did not meet the criteria for library preparation and were excluded.

The pooled amplicons were diluted to 100-200 ng/µl for library preparation (Illumina DNA Prep kit) per the manufacturer’s instructions. Paired-end sequencing was performed on the Illumina NextSeq 550 platform using the v2.5, 300 cycle kit (Illumina, San Diego, CA).

| **Organism** | **Plex** | **Primer Name** | **Sequence (5’-3’)** | **Position (nt)** | **PCR Amplicon Size (bp)** |
| --- | --- | --- | --- | --- | --- |
| **Human metapneumovirus [3]**  (GenBank Accession KU821121) | PCR1 | HMPV1_F | GGGACAAATAAAAATGTCTCTTCA | 41 | 4125 |
|  |  | HMPV1_R | CTTCCTGTGCTRACYTTRCA | 4165 |  |
|  | PCR2 | HMPV2_F | ACAGCAGCRGGRATYAATGT | 4074 | 4010 |
|  |  | HMPV2_R | TAGTACTGAAYTGAGCATGYTCAG | 8083 |  |
|  | PCR3 | HMPV3_F | AACTGTTAACATGGAAAGATGTGATG | 7823 | 3229 |
|  |  | HMPV3_R | TAAGCTGGAACWGAWGCTG | 11,051 |  |
|  | PCR4 | HMPV4_F | TCAATAGGGAGTCTRTGTCARGAA | 9730 | 3675 |
|  |  | HMPV4_R | GRCAAAAAAACCGTATACATYC | 13,404 |  |
| **Respiratory Syncytial Virus [4]**  (GISAID Accession 2,584,506) | PCR1 | RSV1_1F | ACGCGAAAAAATGCGTACTACAAAC | 1 | 1898 |
|  |  | RSV1_1R | CTGMACCATAGGCATTCATAAACA | 1898 |  |
|  |  | RSV1_3F | GCTATGGCAAGACTYAGGAATG | 2926 | 3379 |
|  |  | RSV1_3R | TTGAGRTCTAACACTTTGCTGGT | 6304 |  |
|  |  | RSV1_5F | TGATGCATCAATATCTCAAGTCA | 7181 | 3936 |
|  |  | RSV1_5R | GRCCTATDCCTGCATACTC | 11,116 |  |
|  | PCR2 | RSV2_2F | ATGGGAGARGTRGCTCCAGAATA | 1562 | 2443 |
|  |  | RSV2_2R | CGTGTAGCTGTRTGYTTCCAA | 4,004 |  |
|  |  | RSV2_4F | AGCAAATTYTGGCCYTAYTTTAC | 4334 | 3634 |
|  |  | RSV2_4R1 | CTCATAGCAACACATGCTGATTG | 7967 |  |
|  |  | RSV2_4R2 | GAGTTTGCTCATGGCAACACAT | 7974 | 3641 |
|  |  | RSV2_6F | TGGACCATWGAAGCYATATCA | 10912 | 4354 |
|  |  | RSV2_6R | AGTGTCAAAAACTAATRTCTCGT | 15265 |  |

**Supplementary Table 4.** Information on primers used for multiplex PCR for human metapneumovirus (HMPV) and respiratory syncytial virus (RSV) **(adapted from Tullock et al.,2021 and Dong et al.,2023)**

**References**

1. Shu, B., et al., *Multiplex Real-Time Reverse Transcription PCR for Influenza A Virus, Influenza B Virus, and Severe Acute Respiratory Syndrome Coronavirus 2.* Emerg Infect Dis, 2021. **27**(7): p. 1821-1830.

2. Ng, K.T., et al., *Performance of a Taqman Assay for Improved Detection and Quantification of Human Rhinovirus Viral Load.* Sci Rep, 2016. **6**: p. 34855.

3. Tulloch, R.L., et al., *An Amplicon-Based Approach for the Whole-Genome Sequencing of Human Metapneumovirus.* Viruses, 2021. **13**(3).

4. Dong, X., et al., *A simplified, amplicon-based method for whole genome sequencing of human respiratory syncytial viruses.* J Clin Virol, 2023. **161**: p. 105423.
